# Is Serum Total LDH Evaluation Able to Differentiate between Alimentary Lymphoma and Inflammatory Bowel Disease in a Real World Clinical Setting?

**DOI:** 10.1371/journal.pone.0151641

**Published:** 2016-03-17

**Authors:** Rossella Terragni, Antonio M. Morselli-Labate, Massimo Vignoli, Enrico Bottero, Barbara Brunetti, Jimmy H. Saunders

**Affiliations:** 1 “Petcare” Veterinary Association, Marzabotto, (BO), Italy; 2 Department of Medical and Surgical Sciences, Alma Mater Studiorum - University of Bologna, Bologna, Italy; 3 Department of Veterinary Medical Sciences, Alma Mater Studiorum - University of Bologna, Bologna, Italy; 4 Faculty of Veterinary Medicine, University of Teramo, Piano d'Accio, (TE), Italy; 5 “Argentina” Polyclinic, Arma di Taggia, (IM), Italy; 6 Medical Imaging, Faculty of Veterinary Medicine, Ghent University, Ghent, Belgium; Colorado State University, UNITED STATES

## Abstract

**Context:**

An increase in enzyme lactate dehydrogenase (LDH) in serum is a negative prognostic factor for survival in cats affected by lymphoma. Measuring LDH at the time of diagnosis has been studied for differentiating neoplastic disease from non-neoplastic disease in dogs. Inflammatory bowel disease (IBD) and alimentary lymphoma are common diseases in cats.

**Objective:**

The aim of this study was to determine whether elevation of total LDH occurred in cats with alimentary lymphoma and non-neoplastic gastrointestinal disease, such as IBD, and to evaluate whether this enzyme is useful in supporting the differential diagnosis of these specific diseases.

**Materials and Methods:**

A prospective non-randomized controlled study was carried-out in a real world setting of three Italian private veterinary clinics. Seventy-one client-owned cats with a history of chronic gastrointestinal symptoms were enrolled; 33 cats were histologically diagnosed as having alimentary lymphoma and 38 cats as having IBD. Serum samples of total LDH analysis were measured.

**Results:**

Gender (P = 0.016) and age (P = 0.046) were found to be significant factors influencing the differentiation of serum total LDH between cats with alimentary lymphoma and those with IBD. Despite low diagnostic accuracy in the overall population (63%), a cut-off value of serum total LDH ranging from 0.85- to 1.04-times the upper reference limit showed good capability (accuracy >80%) of differentiating these two conditions in neutered males and cats younger than 8 years of age (AUC: 0.805, 0.833; sensitivities: 76.9%, 83.3%; specificities: 80.0%, 76.5%; PPV: 76.9%, 55.6%; NPV: 80.0%, 92.9%; respectively).

**Conclusions:**

Although our study showed that gender and age are significant factors in differentiating serum total LDH between cats with alimentary lymphoma and those with IBD, this test had poor diagnostic accuracy in differentiating between these two conditions in the overall population.

## Introduction

Lactate dehydrogenase (LDH) is an enzyme which catalyzes the last step of the conversion of pyruvate to lactate during anaerobic glycolysis. Lactate dehydrogenase is found in the cells of almost all body tissues. There are five isoenzymes with LDH activity. Different normal human tissues contain different patterns of these five isoenzymes [1]. Alteration in LDH isoenzyme levels has been observed during development, under changing biological conditions and in response to pathological processes. Lactate dehydrogenase activity in serum increases as a marker of cellular necrosis [2].

Serum LDH and its isoenzyme have been studied extensively in various body cancers. Malignant tumor tissue or contiguous tissue damaged by a tumor liberates enzymes into circulation which contribute towards an abnormal increase in enzyme levels [3]. Regardless of tissue origin, studies on LDH distribution in different types of malignant tumors have shown LDH to be abnormally increased [3]. Moreover, LDH has also been identified as a valuable predictive and/or prognostic biomarker for different types of carcinoma [4]. Serum LDH is commonly increased in patients with hematopoietic malignancies, such as Hodgkin’s lymphoma, non-Hodgkin’s lymphoma or multiple myeloma [5]. Lactate dehydrogenase is one of the risk factors included in the International Prognostic Index, and it is considered a strong predictor of survival in patients with aggressive lymphoid cancers [6]. The total LDH level in the blood may be a meaningful diagnostic parameter in human cancer patients, but the clinical significance of serum isoenzymatic patterns of LDH is still under discussion; the LDH-5 isoform was added to the list of highly promising targets for cancer treatment [5].

In veterinary medicine, LDH has been evaluated in dogs, and it has been demonstrated that LDH levels have limited use in differentiating dogs with cancer from both healthy dogs and dogs with non-neoplastic diseases, as well as in differentiating among different types of tumors [7]. On the other hand, the determination of LDH activity may help in identifying episodes of recurrence in dogs with lymphoma [8]. In feline oncology, it has been reported that an increase in serum LDH activity in the initial chemistry profile was a negative prognostic indicator for survival in cats with lymphoma [9]. As regards isoenzymes, it has been reported that LDH-5 is dominant and LDH-1 is very scant in feline leukocytes, thus suggesting that the leukocytes of cats tend to produce more energy under anaerobic conditions compared to the leukocytes of dogs or rabbits [10].

Lymphomas are a common malignancy affecting cats [11], representing 30% of feline tumors [12]. Alimentary lymphoma (AL) is the most common form of lymphoma in cats [13]. A majority of the T-cell immunophenotype (83%) in feline gastrointestinal lymphoma suggests that previous studies may have underestimated the incidence due to the difficulty in distinguishing mucosal T-cell lymphoma from lymphoplasmacytic inflammatory bowel disease (IBD) [12]. The clinical presentation of feline AL is quite similar to feline IBD [14], a chronic enteropathy characterized by persistent or recurrent gastrointestinal symptoms and histologically confirmed inflammation [15].

The aims of this study were: 1) to determine whether elevation of total LDH occurred in cats with AL and non-neoplastic gastrointestinal diseases, such as IBD, and 2) to evaluate whether this enzyme is useful in supporting the differential diagnosis of these specific diseases.

## Materials and Methods

### Experimental Design

All consecutive client-owned cats with a history of chronic gastrointestinal symptoms, such as weight loss, anorexia, vomiting, and small bowel diarrhea, referred to three Italian private veterinary clinics (“Giardini Margherita” Veterinary Clinic, Bologna; “Modena Sud” Veterinary Clinic, Spilamberto, Modena;”Argentina” Polyclinic, Arma di Taggia, Imperia) were selected for a prospective non-randomized controlled study carried out between January 2013 and September 2015.

The serum LDH levels were compared between AL and IBD cats, and the diagnostic accuracy in differentiating between these two diseases was evaluated. The possible effect of breed, gender, age and body weight was also taken into account.

All owners gave their oral informed consent for the enrollment of their cats into the study. The study protocol conforms to the ethical guidelines of the "World Medical Association Declaration of Helsinki—Ethical Principles for Medical Research Involving Human Subjects". All animals were treated according to the criteria outlined in the "Guide for the Care and Use of Laboratory Animals" of the National Institutes of Health. Ethical approval was not required since no animal research was involved in the study and the data were collected from the records of cats managed according to the usual clinical practice in a real world setting of three veterinary centers.

### Selection Criteria

All cats underwent a physical examination, fecal flotation assays, fecal enzyme-linked immunosorbent assay (ELISA) immunoassay for *Giardia*, a complete blood count, a biochemistry profile, urinalysis, FIV-FeLV serologic ELISA test and serum total T_4_. Abdominal ultrasonography was performed in all patients to exclude non-gastrointestinal causes for their clinical symptoms and to study the gastrointestinal features.

### Exclusion Criteria

Cats which responded to treatment for Giardiasis (fenbendazole 50 mg/kg *per os* once a day for 5 days) or to specific diets (hydrolyzed protein diet, etc.) were excluded in order to exclude adverse food reaction. Patients with primary renal, hepatic or pancreatic disease, hyperthyroidism, diabetes, toxic causes or other intestinal parasitic diseases as well as patients which were highly suspected for cancer (i.e. clinical symptoms of abdominal lesion, such as a palpable mass, lymphadenopathy appreciable on palpation, etc.) were also excluded from the study.

### Diagnostic Procedures

All the cats underwent abdominal ultrasound examination and endoscopic gastrointestinal biopsy to collect stomach and duodenum histological samples in order to differentiate between the AL and the IBD groups. Endoscopies were performed by two endoscopists (R.T. and E.B.). Ileoscopy and ileum biopsy were not routinely performed in the centers involved in the study.

A histopathological diagnosis of all bioptic samples was carried out according to the histopathological standards of gastrointestinal inflammation of endoscopic bioptic samples published by the World Small Animal Veterinary Association (WSAVA) Gastrointestinal Standardization group [16, 17]. The IBD cases were classified as mild, moderate and severe [18]. Cases of lymphomas were graded applying the modified World Health Organization (WHO) classification for lymphoma [19, 20] according to the following method. Lymphomas with 0 to 5 mitoses/400x field were classified as low grade, those with 6 to 10 mitoses/400x field were classified as medium grade and those with greater than 10 mitoses/400x field were classified as high grade [14]. Immunohistochemistry was required if it was needed to confirm the presence of gastrointestinal lymphoma.

### Immunohistochemistry

Two sections 4 μm-thick were cut from the paraffin blocks and labeled by immunohistochemistry with antibodies anti-CD3 (clone CD3-12, Leukocyte Antigen Biology Laboratory, UC Davis School of Veterinary Medicine, Davis, CA, USA) and anti-CD79 (clone HM57, Santa Cruz Biotechnology, Inc., Dallas, TX, USA). The sections were dewaxed in toluene and rehydrated. Endogenous peroxidase was blocked by immersion in H_2_O_2_ 0.3% in methanol for 30 min. The sections were then rinsed in Tris buffer, and the antigen was retrieved with citrate buffer (pH 6.0, CD3 sections) and with ethylenedinitrilotetraacetic acid (EDTA) buffer (pH 8.0, CD79 sections) by heating for two 5-min periods in a microwave at 750 W, followed by cooling at room temperature for 20 min. The antibodies CD3 and CD79 were diluted in phosphate buffered saline (PBS) 1:20 and 1:750, respectively. All antibodies were incubated with the tissue sections overnight at 4°C. Binding sites were revealed using a secondary biotinylated antibody (dilution 1:200) and amplified using a commercial avidin-biotinperoxidase kit (VECTASTAIN ABC Kits, Vector Laboratories, Ltd., Peterborough, UK). Chromogen DAB (3,30diaminobenzidine; 0.05% for 3 minutes at room temperature) was used. The slides were counterstained with Papanicolaou hematoxylin. The primary antibody was replaced with an irrelevant, isotype-matched antibody as a negative control. Reactive lymph nodes were used to assess the specificity of the immunohistochemical procedure.

### LDH Evaluation

The serum total LDH was evaluated at the time of clinical presentation after 12 hours of fasting in all cats. Blood was taken from the jugular vein and the serum was obtained by centrifugation after 20 minutes.

A photometric method for assessing total LDH was carried out in one laboratory (Olympus analyzer; IDEXX Laboratories S.r.l., Milan, Italy; reference range: 0–182 U/L; intra- and inter-assay coefficients of variation (CVs): unavailable) while a kinetic optimized method (SCE, LDH-P; BT1500 VET; Futurlab S.r.l., Limena, PD, Italy; reference range: 63–273 U/L; intra- and inter-assay CVs: 1.69–3.86% and 1.26–2.13%, respecively) was used in the other two laboratories.

Since serum total LDH was evaluated in different laboratories which adopted different reference ranges of the values measured in U/L, these values were standardized and reported as fraction of the upper reference limit (xURL) according to the following formula:

Standrdized total LDH (xURL) = Total LDH (U/L) / Upper reference limit (U/L);

thus, a standardized value equal to 0 xURL corresponded to 0 U/L while a standardized value of 1 xURL corresponded to the upper limit of the reference ranges.

### Statistics

Means, standard deviations, range and frequencies were reported as descriptive statistics. Age and weight were described as scale variables and were also dichotomized for analysis: a cut-off value of 8 years was chosen for age [21, 22] while weight was dichotomized according to the median value (4 kg).

The Fisher’s exact, the Pearson’s chi-square and the linear-by-linear chi-square tests were applied to analyze the dichotomous, nominal and ordinal discrete variables, respectively, while the analysis of variance (ANOVA) was applied in order to compare scale variables between the two groups studied. Serum total LDH values, age and body weight showed distributions significantly different from the normal distribution (P<0.001 for LDH and body weight; P = 0.003 for age) with the Shapiro-Wilks test. The log(x–k) transformation was applied before the analysis of serum total LDH values and body weight since these variables had positive skewness while the exp(x/k) transformation was applied to age since it had negative skewness. In order to reach distributions not significantly different from normality the values of the constant coefficients (k) which zeroed the skewness were chosen for body weight (P = 0.227) and age (P = 0.062) while there was no need for a constant in transforming the LDH values (P = 0.126). One-way ANOVAs were used in order to compare LDH value, age and body weight between AL and IBD cats as well as to compare LDH values among the different histological characteristics of AL and IBD cats. Two-way ANOVAs were also applied in order to analyze the LDH value by adjusting for possible confounding factors (breed, gender, age and body weight). Nested ANOVA designs were used in the two-way ANOVAs in order to test the differences between the different categories of the factors tested within the two groups of cats as well as to test the differences between the two groups of cats within the different categories of the factors tested. The interactions between these factors and the groups were also taken into account in the two-way ANOVAs in order to test whether the differences in LDH observed between the two groups were significantly related to the presence of the various factors.

The diagnostic accuracy of standardized serum total LDH values in differentiating AL from IBD cats was evaluated by means of the area (AUC) under the receiver operating characteristics (ROC) curve. The standard error (SE) of the AUC was computed by means of a distribution-free non-parametric method while the best cut-off value of standardized serum total LDH was identified by means of a maximum likelihood ratio (LR) method according to the following formula: LR = (Frequency of true positive + Frequency of true negative) / (Frequency of false positive + Frequency of false negative) [23]. Sensitivity, specificity, positive predictive value (PPV) and negative predictive value (NPV) were evaluated using the best cut-off value.

Data were managed and analyzed by means of the IBM SPSS Statistics package (version 23.0; IBM Co., Armonk, NY, USA) and a two-tailed P value equal to 0.05 was chosen as the limit of statistical significance.

## Results

### Patients

Seventy-one client-owned cats were used: 64 European shorthair cats, 2 Birman cats, 2 Maine coons, 2 Norwegian forest cats and 1 Persian. Twenty-eight (39.4%) were neutered males and 43 (60.6%) were spayed females; mean age was 9.2±3.8 years, range: 1–16 years (n = 48, 67.6% were over 8 years of age) and the mean weight was 4.1±1.6, range: 2–10 kg (n = 37, 52.1% weighed more than 4 kg).

Abnormalities detected on physical examination included poor body condition (body condition score, BSC: 1-2/5) (n = 32; 45.1%), poor coat condition (n = 35; 49.3%), lethargy (n = 15; 21.1%), diffusely thickened intestinal loops (n = 21; 29.6%), and gaseous or liquid intestinal matter (n = 21; 29.6%). Fecal flotation assays and fecal ELISA immunoassays for the detection of *Giardia* were negative in all cases.

Clinicopathological abnormalities included mild anemia (n = 24; 33.8%), mild neutrophilia (n = 21; 29.6%) and mild hypoalbuminemia (n = 9; 12.7%) as well as mild elevations of azotemia (n = 12; 16.9%), bilirubin (n = 10; 14.1%) and liver enzymes (n = 7; 9.9%). All the cats had a negative serologic ELISA test for retrovirus except for one which was FeLV positive (1.4%). Serological T_4_ was normal in all cats.

### Study Groups

Thirty-three cats had a diagnosis of AL (46.5%) and 38 cats a diagnosis of IBD (53.5%). Of the 33 AL cats, 19 were low grade (57.6%) and 14 were high grade (42.4%) (no cats with intermediate grade were diagnosed). The lymphoma was located in the stomach in 18 out of 33 cats (54.5%), in the small bowel in 11 cats (33.3%) and in both organs in 4 cats (12.1%). Immunophenotyping on histological samples was available in 28 AL cats (84.8%); T-lymphoma was present in 18 of these cats (64.3%) and B-lymphoma was present in 10 cats (35.7%).

Of the 38 IBD cats, 8 (21.1%) had mild disease, 16 had moderate disease (42.1%) and 14 had severe disease (36.8%).

Ultrasonographic examination revealed moderate to severe gastrointestinal wall thickening together with loss of layering in the 14 cats with high grade lymphoma, mild increased wall thickening (muscular layer) with normal layering in 25 cases (14 cases with low grade lymphoma 73.7%; 11 cases with IBD, 28.9%), and normal wall thickening and normal layering in 5 cats with low grade lymphoma (26.3%) and 27 cases with IBD (71.1%). Mild to moderate mesenteric lymph node enlargement was also visible in 32 cats, 25 with lymphoma (75.8%) and 7 with IBD (18.4%).

The upper gastrointestinal endoscopy of the cats with high-grade lymphoma showed an abnormal appearance of the gastric mucosa in 12 cases (85.7%; there was decreased distensibility of the gastric body -also after insufflation-, mild to moderate hyperemia, increased granularity and friability) and focal or linear erosion or ulcers in 7 cases (50.0%). No relevant alterations of the gastric mucosa were found in low-grade lymphoma and IBD cats. In the small bowel of all the cats, the mucosa was friable and irregular, with increased graininess and coerced villi.

The characteristics of the two study groups are shown in Table 1. Breed, gender and body weight were not significantly different between the two groups while the AL cats were more than 2 years older than the IBD cats (P = 0.058); in fact, the majority of them were over 8 years of age (n = 27, 81.8% *vs*. n = 21, 55.3%; P = 0.023).

**Table 1:** Characteristics of the two groups studied.

	All cases (n = 71)	Alimentary lymphoma (n = 33)	IBD (n = 38)	*P value*
**Severity of the disease:**	-	19 (57.6%) Low grade	8 (21.1%) Mild	*-*
		14 (42.4%) High grade	16 (42.1%) Moderate	
			14 (36.8%) Severe	
**Breed:**				*P = 0*.*113* ^a^
- European shorthair	64 (90.1%)	32 (97.0%)	32 (84.2%)	
- Others	7 (9.9%)	1 (3.0%)	6 (15.8%)	
**Gender:**				*P = 1*.*000* ^a^
- Neutered males	28 (39.4%)	13 (39.4%)	15 (39.5%)	
- Spayed females	43 (60.6%)	20 (60.6%)	23 (60.5%)	
**Age (years):**				
- Mean±SD (range)	9.2±3.8 (1–16)	10.3±2.6 (3–16)	8.2±4.5 (1–16)	*P = 0*.*058* ^b^
- >8 years	48 (67.6%)	27 (81.8%)	21 (55.3%)	*P = 0*.*023* ^a^
**Weight (kg):**				
- Mean±SD (range)	4.1±1.6 (2.0–10.0)	4.3±1.7 (2.2–10.0)	4.0±1.5 (2.0–9.8)	*P = 0*.*295* ^b^
- 4 kg or more	37 (52.1%)	17 (51.5%)	20 (52.6%)	*P = 1*.*000* ^a^

^a^ Fisher’s exact test.

^b^ One-way ANOVA.

### Distribution of LDH in AL and IBD Cats

**Fig 1:**
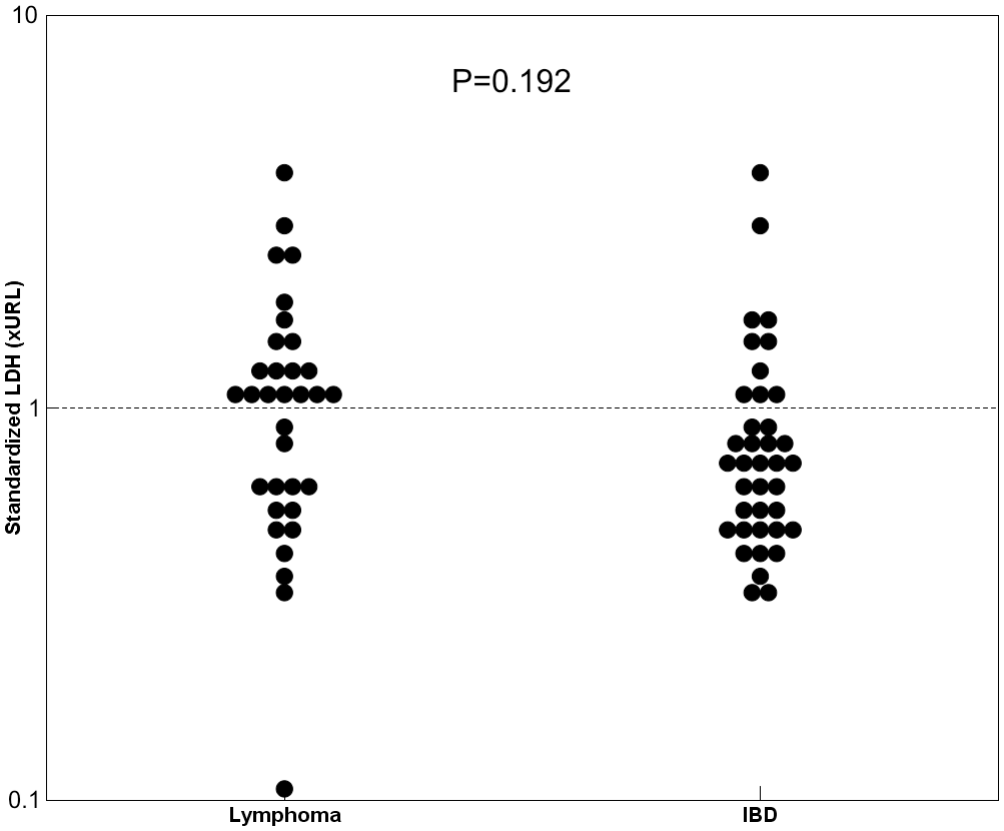
Distribution of standardized serum total LDH values in alimentary lymphoma and IBD cats. Data are reported as fold of the upper reference limit (xURL).

The distribution of the standardized serum total LDH values observed in AL and IBD cats is shown in Fig 1; the descriptive statistics, stratified according to the characteristics of AL and IBD cats, are reported in Table 2. Mean standardized serum total LDH values were higher in cats with lymphoma than in those with IBD (1.16±0.80 in AL *vs*. 0.91±0.73 in IBD), although the unadjusted comparison was not statistically significant (P = 0.192). On the other hand, a significantly (P = 0.009) higher frequency of cats with serum total LDH values higher than the upper reference limit was found in AL cats (n = 19, 57.6%) than in IBD cats (n = 10, 26.3%).

**Table 2:** Descriptive statistics of standardized serum total LDH values (xURL) according to the characteristics of alimentary lymphoma and IBD.

	Descriptive statistics			Abnormal values
	Mean±SD	Median (IQR)	Range	
**Alimentary lymphoma (n = 33)**	**1.16±0.80**	**1.07 (0.59–1.35)**	**0.11–3.84**	**19 (57.6%)**
**Location:**				
- Stomach (n = 18)	1.16±0.79	1.12 (0.62–1.32)	0.11–3.84	12 (66.7%)
- Small bowel (n = 11)	1.36±0.90	1.07 (0.62–2.34)	0.42–2.93	6 (54.5%)
- Stomach and small bowel (n = 4)	0.63±0.32	0.56 (0.36–0.97)	0.33–1.07	1 (25.0%)
	*P = 0*.*295* ^a^			*P = 0*.*303* ^b^
**Immunophenotype:**				
- T-lymphoma (n = 18)	1.03±0.74	0.95 (0.46–1.20)	0.11–2.93	9 (50.0%)
- B-lymphoma (n = 10)	1.15±0.34	1.24 (0.95–1.32)	0.56–1.70	8 (80.0%)
	*P = 0*.*227* ^a^			*P = 0*.*226* ^c^
**Grade:**				
- Low grade (n = 19)	1.02±0.72	0.89 (0.48–1.10)	0.11–2.93	9 (47.4%)
- High grade (n = 14)	1.35±0.90	1.24 (0.63–1.48)	0.46–3.84	10 (71.4%)
	*P = 0*.*155* ^a^			*P = 0*.*286* ^c^
**IBD (n = 38):**	**0.91±0.73**	**0.71 (0.51–1.05)**	**0.35–4.18**	**10 (26.3%)**
**Grade:**				
- Mild (n = 8)	0.65±0.23	0.58 (0.46–0.84)	0.38–1.06	1 (12.5%)
- Moderate (n = 16)	1.02±1.03	0.67 (0.49–0.87	0.35–4.18	3 (18.8%)
- Severe (n = 14)	0.94±0.46	0.76 (0.51–1.44)	0.36–1.66	6 (42.9%)
	*P = 0*.*456* ^a^			*P = 0*.*095* ^d^

xURL: fraction of the upper reference limit.

^a^ One-way ANOVA.

^b^ Pearson chi-square.

^c^ Fisher’s exact test.

^d^ Liner-by-linear chi-square.

Although no significant relationships were found between serum total LDH values and the characteristics of both AL and IBD cats (Table 2), higher total LDH values were found in cats with high grade alimentary lymphoma (1.35±0.90 *vs*. 1.02±0.72 in low grade; P = 0.155) and in those with B-lymphoma (1.15±0.34 *vs*. 1.03±0.74 in T-lymphoma; P = 0.227) as well as a non significant higher frequency of abnormal LDH values was found in cats with severe IBD (42.9% *vs*. 12.5% and 18.8% in mild and moderate IBD, respectively; P = 0.095).

The overall comparison of serum total LDH values between the two groups of cats was significant after adjusting for breed (P = 0.017) and age (P = 0.038) while it did not reach the level of significance after adjusting for gender (P = 0.065) and body weight (P = 0.213); thus showing that breed and age represent important confounding factors and that the stratification of cats according to these factors plays an important role in differentiating serum total LDH values between AL and IBD cats. Table 3 shows the detailed results of the analyses carried out within the various classes of breed, gender, age and body weight. In AL cats, the European shorthair cats (P = 0.023) and the spayed females (P = 0.004) had significantly lower serum total LDH values than the other breeds and neutered males, respectively, while no significant differences were found for breed, gender, age and body weight in IBD cats. Gender (P = 0.016) and age (P = 0.046) had a significant effect in differentiating serum total LDH values between AL and IBD cats; in fact, significantly higher values of LDH in AL *versus* IBD cats were found in neutered males (P = 0.007) and cats up to 8 years of age (P = 0.018) while no significant differences between AL and IBD cats were found in spayed females (P = 0.634) and cats aged over 8 years of age (P = 0.934). In addition, although significantly higher values of LDH in AL than in IBD were found only in breeds other than the European shorthair (P = 0.028) and in cats weighing more than 4 kg (P = 0.028), the effects of breed and weight in the differentiation between AL and IBD only approached the limit of significance (P = 0.059 and P = 0.068, respectively).

**Table 3:** Comparison of standardized serum total LDH values (xURL) between alimentary lymphoma and IBD cats (two-way ANOVAs adjusted for possible confounding factors).

Tested factors	Alimentary lymphoma (n = 33)	IBD (n = 38)	P value
**Breed** (All cases):			
- European shorthair (n = 64)	1.08±0.66 (n = 32)	0.85±0.52 (n = 32	0.253
- Others (n = 7)	3.84 (n = 1)	1.26±1.45 (n = 6)	0.028
*P value*	*0*.*023*	*0*.*592*	*0*.*059* ^a^
**Gender** (All cases):			
- Neutered males (n = 28)	1.62±1.00 (n = 13)	0.93±0.96 (n = 15)	0.007
- Spayed females (n = 43)	0.87±0.47 (n = 20)	0.91±0.56 (n = 23)	0.634
*P value*	*0*.*004*	*0*.*657*	*0*.*016*^a^
**Age** (All cases):			
- Up to 8 years (n = 23)	1.64±1.13 (n = 6)	0.80±0.61 (n = 17	0.018
- Over 8 years (n = 48)	1.06±0.70 (n = 27)	1.00±0.82 (n = 21)	0.934
*P value*	*0*.*082*	*0*.*309*	*0*.*046* ^a^
**Weight** (All cases):			
- Less than 4 kg (n = 34)	0.95±0.49 (n = 16)	1.01±0.63 (n = 18)	0.681
- 4 kg or more (n = 37)	1.36±0.99 (n = 17)	0.83±0.81 (n = 20)	0.028
*P value*	*0*.*152*	*0*.*249*	*0*.*068* ^a^

xURL: fraction of the upper reference limit.

^a^ The P values of the interactions test whether the differences observed between the two groups are significantly different between the classes of the factors tested.

### Diagnostic Accuracy

**Fig 2:**
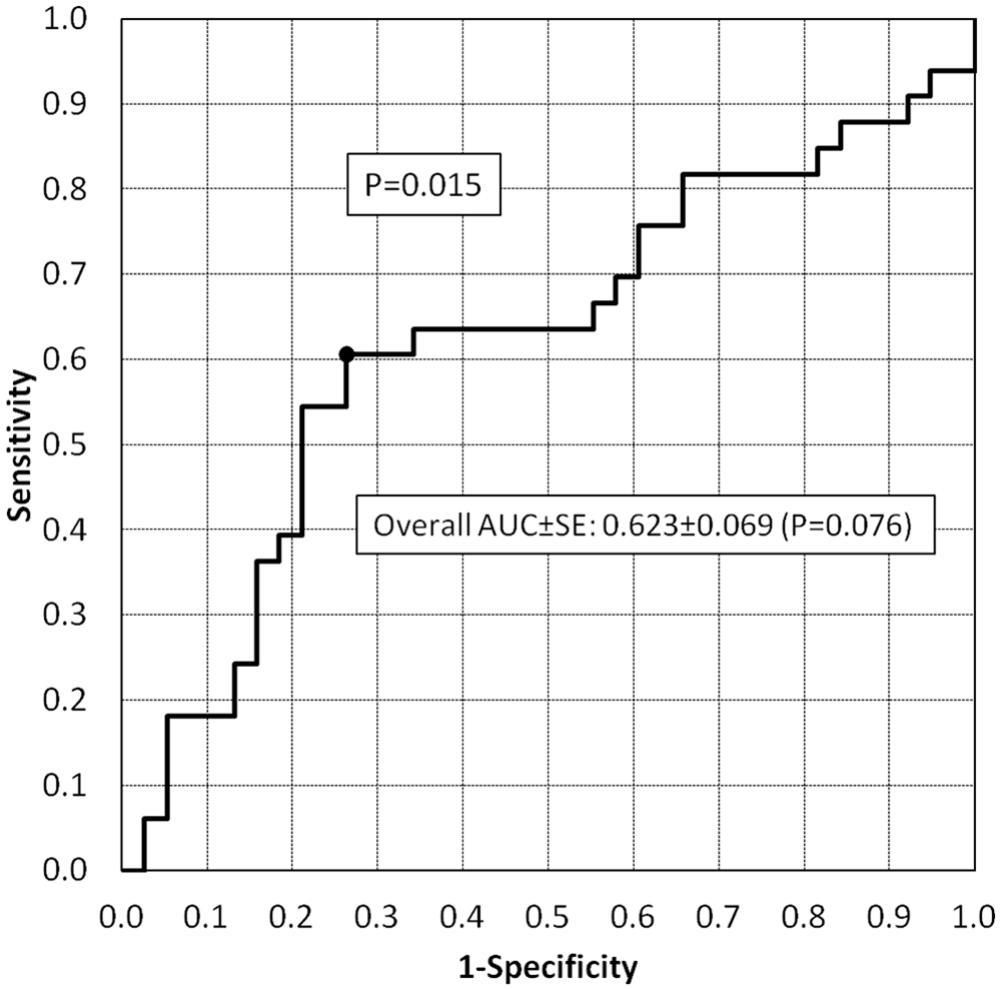
Receiver operating characteristics (ROC) curve of standardized serum total LDH values in differentiating AL from IBD cats. The area under the curve is reported together with the standard error (AUC±SE). The best cut-off (corresponding to a standardized serum total LDH value ranging 0.88–0.89 xURL with a LR value equal to 2.04) is identified in the ROC curve.

The ROC analysis showed poor overall diagnostic accuracy (AUC±SE = 0.623±0.068) which failed to reach the level of significance (P = 0.076; Fig 2). The maximum LR value (2.04) identified the best cut-off as ranging from 0.88 to 0.89 xURL with a low sensitivity, specificity, PPV and NPV: 60.6% (20/33), 73.7% (28/38), 66.7% (20/30) and 68.3% (28/41), respectively.

**Fig 3:**
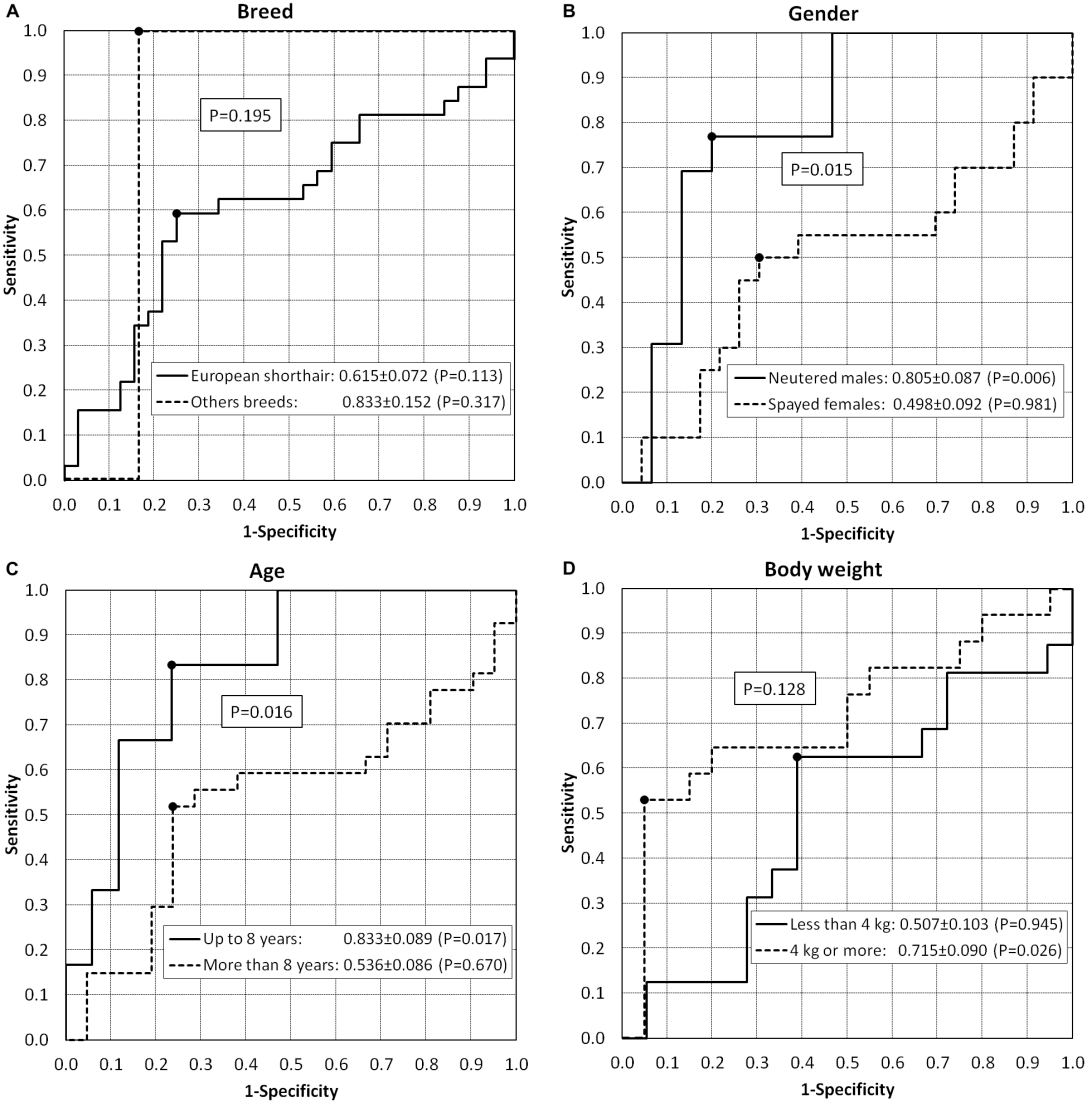
Receiver operating characteristics (ROC) curves of standardized serum total LDH values in differentiating AL from IBD cats stratified according to breed (A), gender (B), age (C), and body weight (D). The area under the curve is reported together with the standard error. The best cut-off values are also identified in the ROC curves.

Fig 3 and Table 4 show the results of the ROC analysis stratified according to the characteristics of the cats studied. Gender (P = 0.015) and age (P = 0.016) significantly conditioned the diagnostic accuracy; in fact, significant values of accuracy were only found in neutered males (80.5%; P = 0.006) and cats up to 8 years of age (83.3%; P = 0.017). In particular, specificity prevailed in neutered males (80.0%) while sensitivity (83.3%) prevailed in younger cats; these figures provided good values of NPV in both groups (80.0% in neutered males and 92.9% in cats up to 8 years of age). It should be pointed out that the high accuracy found in breeds other than the European shorthair (83.3%) failed to reach the level of significance due to the low number of cats. As far as body weight was concerned, the accuracy was only significant in cats weighing more than 4 kg (71.5%; P = 0.026) and was not significantly different (P = 0.128) from the cats weighing less than 4 kg.

**Table 4:** Results of the ROC analysis of standardized serum total LDH values in differentiating AL from IBD cats stratified according to breed, gender, age and body weight.

Factors tested	Accuracy (AUC±SE)	Maximum LR	Best cut-off (xURL)	Sensitivity	Specificity	PPV	NPV
**Breed:**							
- European shorthair	0.615±0.072 (P = 0.113)	2.05	0.88–0.89	19/32 (59.4%)	24/32 (75.0%)	19/27 (70.4%)	24/37 (64.9%)
- Others	0.833±0.152 (P = 0.317)	11.0	1.06–3.84	1/1 (100%)	5/6 (83.3%)	1/2 (50.0%)	5/5 (100%)
	*P = 0*.*195*						
**Gender:**							
- Neutered males	0.805±0.087 (P = 0.006)	3.64	0.85–1.04	10/13 (76.9%)	12/15 (80.0%)	10/13 (76.9%)	12/15 (80.0%)
- Spayed females	0.498±0.092 (P = 0.981)	1.49	0.88–0.89	10/20 (50.0%)	16/23 (69.6%)	10/17 (58.8%)	16/26 (61.5%)
	*P = 0*.*015*						
**Age:**							
- Up to 8 years	0.833±0.089 (P = 0.017)	3.98	0.85–1.04	5/6 (83.3%)	13/17 (76.5%)	5/9 (55.6%)	13/14 (92.9%)
- Over 8 years	0.536±0.086 (P = 0.670)	1.78	1.04–1.06	14/27 (51.9%)	16/21 (76.2%)	14/19 (73.7%)	16/29 (55.2%)
	*P = 0*.*016*						
**Weight:**							
- Less than 4 kg	0.507±0.103 (P = 0.945)	1.62	0.88–0.89	10/16 (62.5%)	11/18 (61.1%)	10/17 (58.8%)	11/17 (64.7%)
- 4 kg or more	0.715±0.090 (P = 0.026)	2.84	1.06–1.07	9/17 (52.9%)	19/20 (95.0%)	9/10 (90.0%)	19/27 (70.4%)
	*P = 0*.*128*						

AUC: area under the curve; LR: likelihood ratio; N/e: not evaluable; NPV: negative predictive value; PPV: positive predictive value; ROC: receiver operating characteristics; SE: standard error; xURL: fraction of the upper reference limit.

## Discussion

Middle-aged and old cats are often referred to the veterinary clinic due to the presence of gastrointestinal symptoms, such as weight loss, anorexia, vomiting and diarrhea. In these patients, the diagnostic plan is complex and frequently leads to gastrointestinal biopsy in order to obtain histological samples for diagnosis. Both IBD and AL are very common pathological conditions in cats, and they present a comparable clinical course [14]. Moreover, in humans, B-cell lymphomas developing after longstanding chronic inflammation seem to be common, and the association between bacterial and viral infections with lymphoma development is well documented for mucosa-associated lymphoid tissue (MALT) B-cell lymphomas [20]. The first step in the clinical evaluation of these patients is non-invasive routine laboratory testing and abdominal ultrasound examination [14]. Despite the fact that moderate to severe wall thickening, together with a loss of layering, presents a poor specificity, it is a highly suggestive ultrasonographic finding of neoplastic lesions (such as high-grade lymphomas); on the other hand, the wall appearance is similar among low grade lymphomas and IBD [24]. In fact, both IBD and AL are characterized by diffuse or segmental distribution in the small intestine, with the ultrasonographic features of bowel wall thickening owing to the increase in the muscularis propria and preservation of the wall layers without mass formation [24]. Moreover, a normal ultrasonographic examination does not rule out AL or IBD. However, in our study, cats with thickening of the muscularis propria detected on ultrasonography and mesenteric lymphadenopathy were more likely to have lymphoma than IBD, and this is in accordance with another recent study [25].

The measurement of LDH as an initial screening for differentiating patients with different cancers (i.e. not only for lymphoma) and patients with non-oncological diseases has been used in dogs, but has not produced convincing results [9]. On the other hand, serum LDH levels demonstrated a prognostic value in canine lymphoma recurrence [11] and an LDH increase was correlated with a worse prognosis in feline lymphoma, similar to the results observed in humans [13]. Therefore, assessing serum total LDH as a possible marker for AL may play an important role in differentiating between these two conditions in cats.

The results of our study showed a significant correlation between high serum total LDH values and AL in cats; in fact, approximately 60% of the AL cats had abnormal LDH values as compared with 26% of the IBD cats. These figures were not significantly related to the location and immunophenotype of the lymphoma or to the grade of both AL and IBD diseases. In addition, serum total LDH levels were significantly related to breed and gender in AL cats while no significant relationships with these factors were found in IBD cats. Thus, the significant differences between AL and IBD were only confirmed in some subgroups of cats (i.e. other breeds than the European shorthair, neutered males, cats younger than 8 years of age and cats weighing more than 4 kg). This finding was suggestive of a possible relationship between these factors and accuracy.

Since the difference in the serum total LDH values observed between AL and IBD cats was statistically significant only after adjusting for some confounding factors (such as breed and age), the diagnostic accuracy in differentiating these two groups of cats in the overall population was low (62%), showing poor sensitivity (61%) and specificity (74%). However, in our study, the subgroups of cats which showed good accuracy of serum total LDH values in differentiating between AL and IBD were identified. These subgroups were neutered males and cats under 8 years of age; they both showed accuracy values of greater than 80%. In particular, a neutered male cat with an LDH value greater than 1.04-times the upper reference limit has a 77% probability of having AL while a neutered male cat with an LDH value less than 0.85-times the upper reference limit has a 80% probability of having IBD. Unfortunately, the range of variation of the best cut-off is quite large due to the low number of cases available. Again, the diagnostic accuracy in cats under 8 years of age showed a PPV higher than 90% at the expense of a low NPV (56%). It has been shown in the literature that cats over 7 years of age have an increased risk of tumors and that intact males and females appeared to have a decreased risk as compared with neutered patients, but this could be explained by the age difference among these patients as the older patients were more likely to be neutered [26]. Possible explanations of our findings might be that LDH levels are more sensitive to the presence of tumors in cats under 8 years of age since they have a lower probability of tumor and it could be hypothesized that slightly underweight cats present increased LDH serum levels similar to the results found in human people with cachexia [27]. Despite these observations, the role that gender and age may play in differentiating AL from IBD cats needs to be analyzed in additional studies.

Our study also showed good discrimination between AL and IBD in breeds other than the European shorthair, but the low number and the heterogeneity of this group of cats limit the strength of this finding. As far as the relationship between breed and risk of tumor is concerned, an increased risk in the Siamese breed has been found [26]. There were no Siamese cats in our population; therefore, a confirmation of the role of LDH in specific breeds is needed involving larger samples. Moreover, additional studies should be aimed at identifying whether the clear difference between AL and IBD serum LDH values found in purebred cats by our group is related to the Siamese or other specific breeds.

The main strength of the present study is the particular selection of cases made in a real-world setting according to a prospective protocol. Fixed previously-defined groups of AL and IBD cats were not studied but only those patients which, at the end of the blood and imaging screening for chronic gastrointestinal symptoms, required biopsy in order to be diagnosed as having intestinal inflammation or lymphoma. Thus, the manifest cases of lymphoma, as well as those with other benign chronic enteropathies already suspected by means of the initial screening, were not included in the study in order to ensure that the results of the present paper could be reliably applied in clinical practice.

The main limitation of the present study was the scarce sample size available due to the strict selection criteria applied. In particular, this weakness prevented the possibility of better identifying the factors which play an independent role in affecting the differentiation between AL and IBD using multivariate analysis. In addition, a larger sample size would also have allowed us to reduce the gray zone of the best cut-off values estimated in some subgroups of cats. Another limitation of the present study was the failure to include the histopathologic evaluation of the ileal bioptic specimens. New studies have now confirmed the need to collect both duodenal and ileal bioptic specimens in dogs and cats having diarrhea caused by intestinal inflammation in either the small or the large intestine (e.g., IBD) or cancer (e.g., lymphoma) [28]. Even though ileal biopsy yields important diagnostic information, ileoscopy is a more technically demanding and time-consuming endoscopic procedure for many clinicians and, for this reason, the new endoscopic recommendations do not include the histopathologic review of ileal mucosal specimens in the initial screening for chronic gastrointestinal symptoms [29].

## Conclusions

Although abnormal serum total LDH values measured at the initial screening were significantly more frequent in AL cats than in IBD cats, this test had poor diagnostic accuracy in differentiating between these two conditions in the overall population. On the other hand, our study showed a good accuracy (greater than 80%) of serum total LDH in differentiating between AL and IBD in neutered males and in cats up to 8 years of age even if the diagnostic utility of LDH may warrant additional studies because of the small size of these subsets of patients.

## Supporting Information

S1 File Source data. (XLSX)

S2 File List of the SPSS (statistical package version 23) commands used for producing all the results reported in the paper. (DOCX)

S3 File List of the results produced by the SPSS package. (XLSX)

## Abbreviations

ANOVA: analysis of variance
AUC: area under the curve
BSC: body condition score
CV: coefficient of variation
LDH: enzyme lactate dehydrogenase
IBD: inflammatory bowel disease
AL: alimentary lymphoma
LR: likelihood ratio
NPV: negative predictive value
PPV: positive predictive value
ROC: receiver operating characteristics
SE: standard error
xURL: fold of upper reference limit
