# Supplementary material for: Is Serum Total LDH Evaluation Able to Differentiate between Alimentary Lymphoma and Inflammatory Bowel Disease in a Real World Clinical Setting?
Source: PLoS One. 2016 Mar 17;11(3):e0151641. doi: 10.1371/journal.pone.0151641 (PMC4795755; doi:10.1371/journal.pone.0151641)
Supplement: S2 File — (DOCX) [file pone.0151641.s002.docx]

* Encoding: UTF-8.

DATASET CLOSE all.

NEW FILE.

***********************************************************************************************.

GET DATA /TYPE=XLSX

/FILE='C:\Users\antonio.morselli\Documents\D\Terragni\LDH\DataALvsIBD.xlsx'

/SHEET=name 'DataALvsIBD'

/CELLRANGE=full

/READNAMES=on

/ASSUMEDSTRWIDTH=32767.

EXECUTE.

************************************************************************************************.

**** SENZA METASTASI (Quindi FNA e BIOSPIE CHIRURGICHE tranne una *******.

**** DROP=0: 21 Metastasi (8 EB, 9 FNA, 4 SB) ***************************************.

**** DROP=0: Si mantiene 1 SB (ID=LM287NA, n=74 **********************************.

**** TOT = 69 casi: 36 IBD + 33 AL ****************************************************.

CROSSTABS stage by Grx by drop / mis = inc .

SELECT IF ( drop = 0 AND ( Stage = 0 OR GRx = 2 ) ) .

CROSSTABS stage by Grx by drop / mis = inc .

*****************************************.

**** Min skewness . .

EXAMINE Weight Ageyears n0LDH / plot = npplot .

compute Ln0LDH = lg10 ( n0LDH ) .

compute EAgeyears = exp ( Ageyears ) .

compute LWeight = lg10 ( Weight ) .

EXAMINE LWeight EAgeyears Ln0LDH / plot = npplot .

compute nAgeyears = exp ( Ageyears / 15.05 ) .

examine nAgeyears / plot = npplot .

compute nWeight = lg10 ( Weight - 1.586 ) .

EXAMINE nWeight / plot = npplot .

***** RESULTS *****************************.

FREQUENCIES Breed iBreed Sex iAgeyears iWeight iTypeofsample .

FREQUENCIES Ageyears Weight / sta = all / for = not.

FREQUENCIES BodyCond CoatCond Lethargy DiffThick IntestMatter Anemia Neutrophilia Hypoalbuminemia Azotaemia Bilirubin LiverEnzymes .

CROSSTABS iFIVFeLV by Grx / cel =cou col /sta = chi .

FREQUENCIES Grx .

FREQUENCIES DIA Location Immunophenotype .

FREQUENCIES IBDseverity.

**** Dati endoscopia fatti a mano **********.

*** TABLE 1 ****.

CROSSTABS DIA idia IBDseverity Breed iBreed Sex iAgeyears iWeight by Grx / cel = cou col / sta = chi .

FREQUENCIES Ageyears Weight / sta = all / for = not.

sort cases by Grx .

SPLIT FILE by Grx .

FREQUENCIES Ageyears Weight / sta = all / for = not.

SPLIT FILE off.

manova nAgeyears by Grx (1,2).

manova nWeight by Grx (1,2).

*** FIG 01 *****.

* manova Ln0LDH by grx ( 1 , 2 ) iBreed ( 0 , 1 ) sex ( 0 , 1 ) iAgeyears ( 1 , 2 ) iWeight ( 1 , 2 )

/DESIGN grx iBreed Sex iAgeyears iWeight

.

GGRAPH

/GRAPHDATASET NAME="graphdataset"

VARIABLES=Ln0LDH[LEVEL=scale] Grx[LEVEL=nominal]

MISSING=LISTWISE REPORTMISSING=NO

/GRAPHSPEC SOURCE=VIZTEMPLATE(NAME="2-D Dot Plot"[LOCATION=LOCAL]

MAPPING( "x"="Grx"[DATASET="graphdataset"] "y"="Ln0LDH"[DATASET="graphdataset"]))

VIZSTYLESHEET="Traditional"[LOCATION=LOCAL]

LABEL='2-D DOT PLOT: Gr-Ln0LDH'

DEFAULTTEMPLATE=NO.

manova Ln0LDH by grx ( 1 , 2 ) .

MEANS n0LDH by Grx .

*** TABLE 2 ****.

SORT CASES by Grx.

SPLIT FILE by Grx.

FREQUENCIES n0LDH / for=not / sta=all / nti = 4 .

SPLIT FILE off.

CROSSTABS Grx by in0LDH / cel = cou row / sta = chi .

sort cases by Location .

SPLIT FILE by Location .

FREQUENCIES n0LDH / for=not / sta=all / nti = 4 .

split file off .

CROSSTABS Location by in0LDH by Grx / cel = cou row / sta=chi ris .

manova Ln0LDH by Location ( 1 , 3 ) .

sort cases by iImmunophenotype .

SPLIT FILE by iImmunophenotype .

FREQUENCIES n0LDH / for=not / sta=all / nti = 4 .

split file off .

manova Ln0LDH by iImmunophenotype ( 1 , 2 ) .

CROSSTABS iImmunophenotype by in0LDH by Grx / cel = cou row / sta=chi ris .

sort cases by iDIA .

SPLIT FILE by iDIA .

FREQUENCIES n0LDH / for=not / sta=all / nti = 4 .

split file off .

manova Ln0LDH by iDIA ( 1 , 2 ) .

CROSSTABS iDIA by in0LDH by Grx / cel = cou row / sta=chi ris .

sort cases by IBDseverity .

SPLIT FILE by IBDseverity .

FREQUENCIES n0LDH / for=not / sta=all / nti = 4 .

split file off .

manova Ln0LDH by IBDseverity ( 1 , 3 )

/DESIGN = IBDseverity .

CROSSTABS IBDseverity by in0LDH by Grx / cel = cou row / sta=chi ris .

**** TABLE 3 *****.

MEANS n0LDH by grx by ibreed .

manova Ln0LDH by grx ( 1 , 2 ) iBreed ( 0 , 1 )

/DESIGN

/DESIGN = grx ibreed within grx (1) ibreed within grx (2)

/DESIGN = ibreed grx within ibreed (2) grx within ibreed (1)

.

MEANS n0LDH by grx by sex .

manova Ln0LDH by grx ( 1 , 2 ) sex ( 0 , 1 )

/DESIGN

/DESIGN = grx sex within grx (1) sex within grx (2)

/DESIGN = sex grx within sex (1) grx within sex (2)

.

MEANS n0LDH by grx by iAgeyears .

manova Ln0LDH by grx ( 1 , 2 ) iAgeyears ( 1 , 2 )

/DESIGN

/DESIGN = grx iAgeyears within grx (1) iAgeyears within grx (2)

/DESIGN = iAgeyears grx within iAgeyears (1) grx within iAgeyears (2)

.

MEANS n0LDH by grx by iWeight .

manova Ln0LDH by grx ( 1 , 2 ) iWeight ( 1 , 2 )

/DESIGN

/DESIGN = grx iWeight within grx (1) iWeight within grx (2)

/DESIGN = iWeight grx within iWeight (1) grx within iWeight (2)

.

**** ACCURATEZZA DIAGNOSTICA ****.

format n0LDH (f8.6).

ROC n0LDH BY Grx (1)

/PLOT=CURVE

/PRINT=SE COORDINATES

/CRITERIA=CUTOFF(INCLUDE) TESTPOS(LARGE) DISTRIBUTION(FREE) CI(95)

/MISSING=EXCLUDE.

CROSSTABS n0LDH by Gr / cel = cou .

recode n0LDH ( lo thru 0.885 = 0 )( 0.885 thru hi = 1 ) into iBestn0LDH_total .

CROSSTABS Gr by iBestn0LDH_total / cel = cou row col .

*** FIGURE 3 ****.

*** FARLA IN Excel ROc-Grafici.xls *******.

sort cases by ibreed .

SPLIT FILE by ibreed .

ROC n0LDH BY Grx (1)

/PLOT=CURVE

/PRINT=SE COORDINATES

/CRITERIA=CUTOFF(INCLUDE) TESTPOS(LARGE) DISTRIBUTION(FREE) CI(95)

/MISSING=EXCLUDE.

split file off .

CROSSTABS n0LDH by Gr by ibreed / cel = cou .

if ( ibreed = 0 AND n0LDH lt 2.00 ) iBestn0LDH_ibreed = 0 .

if ( ibreed = 0 AND n0LDH gt 2.00 ) iBestn0LDH_ibreed = 1 .

if ( ibreed = 1 AND n0LDH lt 0.885 ) iBestn0LDH_ibreed = 0 .

if ( ibreed = 1 AND n0LDH gt 0.885 ) iBestn0LDH_ibreed = 1 .

CROSSTABS Gr by iBestn0LDH_ibreed by ibreed / cel = cou row col .

sort cases by sex .

SPLIT FILE by sex .

ROC n0LDH BY Grx (1)

/PLOT=CURVE

/PRINT=SE COORDINATES

/CRITERIA=CUTOFF(INCLUDE) TESTPOS(LARGE) DISTRIBUTION(FREE) CI(95)

/MISSING=EXCLUDE.

split file off .

CROSSTABS n0LDH by Gr by sex / cel = cou .

if ( sex = 0 AND n0LDH lt 0.90 ) iBestn0LDH_sex = 0 .

if ( sex = 0 AND n0LDH gt 0.90 ) iBestn0LDH_sex = 1 .

if ( sex = 1 AND n0LDH lt 0.885 ) iBestn0LDH_sex = 0 .

if ( sex = 1 AND n0LDH gt 0.885 ) iBestn0LDH_sex = 1 .

CROSSTABS Gr by iBestn0LDH_sex by sex / cel = cou row col .

sort cases by iAgeyears .

SPLIT FILE by iAgeyears .

ROC n0LDH BY Grx (1)

/PLOT=CURVE

/PRINT=SE COORDINATES

/CRITERIA=CUTOFF(INCLUDE) TESTPOS(LARGE) DISTRIBUTION(FREE) CI(95)

/MISSING=EXCLUDE.

split file off .

CROSSTABS n0LDH by Gr by iAgeyears / cel = cou .

if ( iAgeyears = 1 AND n0LDH lt 0.90 ) iBestn0LDH_iAgeyears = 0 .

if ( iAgeyears = 1 AND n0LDH gt 0.90 ) iBestn0LDH_iAgeyears = 1 .

if ( iAgeyears = 2 AND n0LDH lt 1.05 ) iBestn0LDH_iAgeyears = 0 .

if ( iAgeyears = 2 AND n0LDH gt 1.05 ) iBestn0LDH_iAgeyears = 1 .

CROSSTABS Gr by iBestn0LDH_iAgeyears by iAgeyears / cel = cou row col .

sort cases by iWeight .

SPLIT FILE by iWeight .

ROC n0LDH BY Grx (1)

/PLOT=CURVE

/PRINT=SE COORDINATES

/CRITERIA=CUTOFF(INCLUDE) TESTPOS(LARGE) DISTRIBUTION(FREE) CI(95)

/MISSING=EXCLUDE.

split file off .

CROSSTABS n0LDH by Gr by iWeight / cel = cou .

if ( iWeight = 1 AND n0LDH lt 0.885 ) iBestn0LDH_iWeight = 0 .

if ( iWeight = 1 AND n0LDH gt 0.885 ) iBestn0LDH_iWeight = 1 .

if ( iWeight = 2 AND n0LDH lt 1.065 ) iBestn0LDH_iWeight = 0 .

if ( iWeight = 2 AND n0LDH gt 1.065 ) iBestn0LDH_iWeight = 1 .

CROSSTABS Gr by iBestn0LDH_iWeight by iWeight / cel = cou row col .
